# The Animal in Me: Enhancing Emotion Recognition in Adolescents with Autism Using Animal Filters

**DOI:** 10.1007/s10803-019-04179-7

**Published:** 2019-08-26

**Authors:** Liam Cross, Myles Farha, Gray Atherton

**Affiliations:** 1 Department of Psychology, Edge Hill University, Ormskirk, L39 4QP UK; 2 Psychology Department, School of Science, University of Buckingham, Buckingham, MK18 1EG UK

**Keywords:** Autism, Theory of mind, Anthropomorphism, Emotion recognition, Facial processing, Intellectual disability

## Abstract

People with autism are often characterized as having difficulties with theory of mind abilities such as emotion recognition. However, rather than being a pervasive deficit of ‘mindblindness,’ a number of studies suggests these difficulties vary by context, and when people with autism mindread non-human agents, such as animals or cartoons, these abilities improve. To replicate this effect, 15 adolescents with both autism and intellectual disability participated in a test of facial emotion recognition, with both human and animal faces. Participants performed significantly better on the animal version of the assessment compared to the human version, and human rather than animal scores were the strongest predictor of symptom severity. These results were shown to be primarily driven by improvement in recognition of the emotions happiness and anger in animal rather than human faces. Implications with regards to social motivation and theory of mind interventions are discussed.

Theory of mind (ToM), also known as mindreading, refers to the ability to both infer and predict thoughts and emotions in other people by using contextual and interpersonal cues, such as recognizing changes in facial expressions (Premack and Woodruff 1978). A large body of research indicates that people with autism spectrum disorder (ASD) have difficulties with ToM abilities such as emotion recognition (Lozier et al. 2014).

For instance, Sucksmith et al. (2013) looked at differences in facial emotion recognition performance using the Karolinska Directed Emotional Faces test (KDEF) (Lundqvist et al. 1998) between adults with ASD, parents of a child with ASD, and unaffected members of the general population. They found that only participants with ASD showed difficulty identifying emotions, particularly the emotions happy, angry and afraid. This is in line with other research indicating that individuals with ASD have difficulty with these emotions. For instance, Spencer et al. (2011) found differences in neural activation levels between those with ASD and their siblings relative to controls in response to viewing happy faces.

Interestingly, a number of studies suggest that the magnitude of these difficulties vary according to the characteristics of the agent being mindread. Specifically, when autistic people mindread human agents, ToM abilities are at a greater deficit than when mindreading non-human agents with anthropomorphic features, including animals and cartoons (for a review see Atherton and Cross 2018). For example Brosnan et al. (2015) showed that individuals with autism have improved emotion recognition when interpreting the emotions of anthropomorphic (cartoon) versus typically human stimuli. Additionally Atherton and Cross (2019) have also found similar patterns in the broader autism spectrum using non visual, perspective taking ToM task. Interestingly, Golan et al. (2010) showed that anthropomorphising faces (by transposing them on to vehicles) can improve emotion recognition in individuals with ASD, thus highlighting the potential pragmatic avenue to interventions for this work. However the vast majority of this work has been done on individuals with IQ in the typical range, which is not entirely representative of the entire autism spectrum.

Autism is a particularly heterogeneous condition, approximately 55% of those with ASD also have co-occuring intellectual disability and more severe symptomology than those with average IQ scores (Charman et al. 2011). Despite this, the majority of research into autism relies on a high functioning sample with average IQ scores, due to a need for matched control groups (Ozonoff et al. 1990). It is therefore unclear clear whether people with autism who have more severe functional impairments also show improved ToM when agents are non-human. Therefore, the present study explores whether individuals with autism, who are characterized as low functioning, also show improved ToM towards non-human or anthropomorphic agents compared to typically human agents.

## Methods

### Participants and Ethics

Fifteen adolescents from a residential school for individuals with severe autism and intellectual disabilities in the East Midlands area of the UK, aged 12–17 years old, (M age = 15.33, SD = 1.54, four females) participated in the study. The developmental ages of the sample ranged from 4 to 10 years, supplied by occupational therapists at the school. All participants had been diagnosed with autism and more than half also had a formal diagnosis of co-occurring intellectual disability given by their general practitioners. Almost all had a co-occurring mental health diagnosis. Each participant was also assessed using the Gillian Asperger Diagnostic Scale (GADS) (Gilliam 2001) (mean score = 80.07, SD = 8.30, range 70–98). Please see Table 1 for participant characteristics.

**Table 1:** Participant characteristics

Gender	Age	GADS score	Diagnoses
M	12	82	Autism, ADD, Tourette’s
M	15	79	Autism, ID
M	16	79	Autism, ID
M	16	74	Autism
M	16	78	Autism, ADHD, ID
M	16	96	Autism, ADD
M	16	98	Autism
M	16	71	Autism, ID
M	17	71	Autism, ID, ADHD
M	17	77	Autism, ID
M	17	83	Autism, ADHD
F	13	70	Autism, ID
F	13	85	Autism
F	15	75	Autism, ID
F	15	83	Autism

The GADS is a 32-item norm-referenced rating scale. Respondents rate the individual in question on the frequency of ASD indicative behaviors across four subscales: social interaction, restricted patterns of behavior, cognitive patterns, and pragmatic skills. The GADS can not only be used to identify individuals with ASD, but to also document the severity of behaviors, as it can be used to document behavioral progress. The use of the GADS therein is that it is able to both identify the presence of ASD, and qualify the severity of ASD related behaviors. Importantly, testing for the GADS requires responders to be limited to individuals who have had sustained contact with the individual (Campbell 2005), thus strengthening external validity. When recruiting for the study, all students with a diagnosis of autism were verbally invited to take part, and parental consent forms were obtained from the parent/guardian of any individual who expressed interest. Ethical approval was granted by the University of University of Buckingham Ethics Review Board.

### Design, Procedure and Materials

This study employed a within-groups design with one independent variable, Presentation Type, having two levels, Original Human and Animal Filter. This study was run in person, with participants tested individually in a quiet classroom during school hours by the one of the researchers. A single session lasted about 30 min.

As this study required participants to match words with pictures, it was necessary to first confirm that individuals were able to understand both the words and the task. Thus, in the first phase of the study, the experimenter laid out five laminated notecards, each displaying a simple emoji face representing one of the five basic emotions to be tested (Angry, Sad, Afraid, Happy and Surprised). These emojis were also in use therapeutically at the school, and thus familiar to the students. One at a time, the participant was given one laminated notecard upon which one of the same five basic emotion words were written and read a definition and example for the given emotion. The participants were then instructed to place the word card on top of the relevant emoji (order of presentation randomised). All 15 participants passed this check for all five words.

In the second phase, the five emotion word cards used in phase one were laid in front of the participant. They were then told they would be shown several pictures of faces, and their job was to place the correct word on top of the face. After every trial the participant was asked a check question of “Are you sure?” and this was only asked once. If an answer was changed after asking “Are you sure?” then this was the answer that was recorded. This procedure was used because this sample showed difficulty at times with sustained attention, and it could be unclear whether they were sufficiently engaged in the task on their initial choice.

There were twenty emotion pictures used in total, ten of human faces, and the same ten pictures of human faces instead shown in an animal filter. All face pictures were taken from the KDEF and used four different models (two male, two female). Each of the five emotions first tested at baseline were tested twice in each condition (Original Human and Animal Filter), with each emotion displayed with a male and a female face. All face images were pictured head-on, in full color, and filled a full letter size page (8.5 × 11 ins). Please see Table 2 for the items.

**Table 2:**
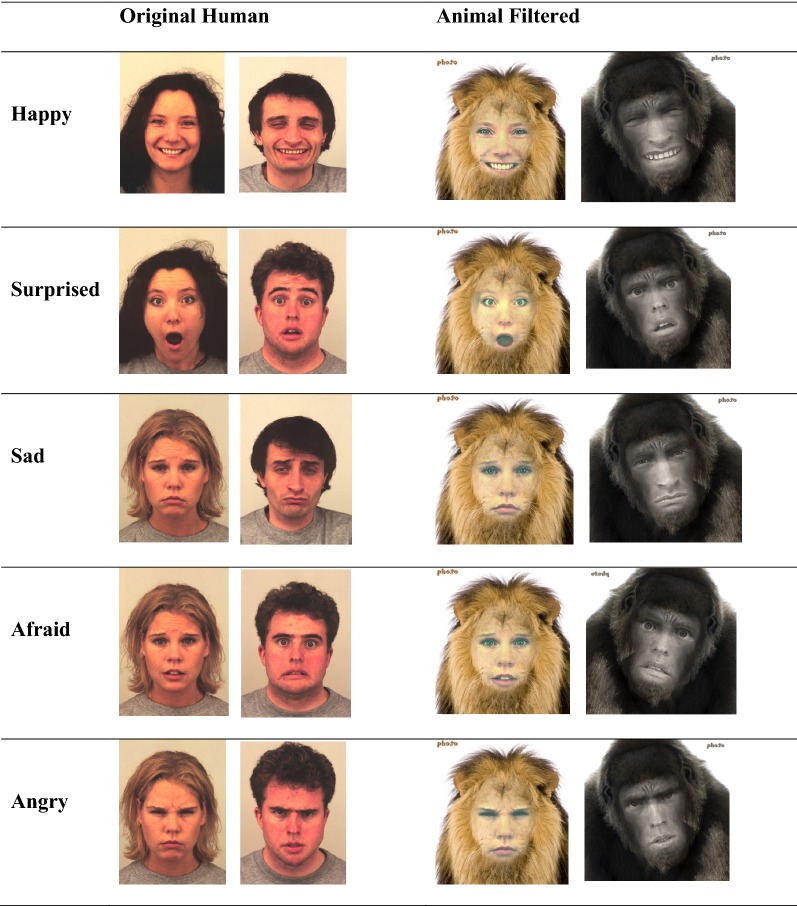
The original and anthropomorphic stimuli

The ten pictures that made up the Original Human emotion set were unaltered from the original KDEF test. For the Animal Filter set, these same ten pictures were put through an animal filter process (retrieved from http://funny.pho.to/human-to-animal-montages/). All female faces were presented in a lion filter, and male faces in a gorilla filter. There were color differences between the human and animal faces, specifically, the animal filters recolored the faces to fit the respective animal theme. However, research suggests that these lower level perceptual differences do not affect emotion recognition (Calvo and Nummenmaa 2011). Importantly, the animal images did not change the inner aspects of the face (aka eye size, mouth positioning), meaning that this filter did not affect higher level face elements that could explain any performance differences. This means that the differences between animal and human faces used in the study are not simply a product of increasing the salience of the high level aspects of the face (aka the eyes) but are instead a factor of the context in which the face/emotion is presented.

Half of the participants saw the Animal Filter set first and the other half the Original Human set first. Within a set the pictures were shown one at a time in random order, and the word cards placed in front of participants were shuffled each round.

## Results

**Fig. 1:**
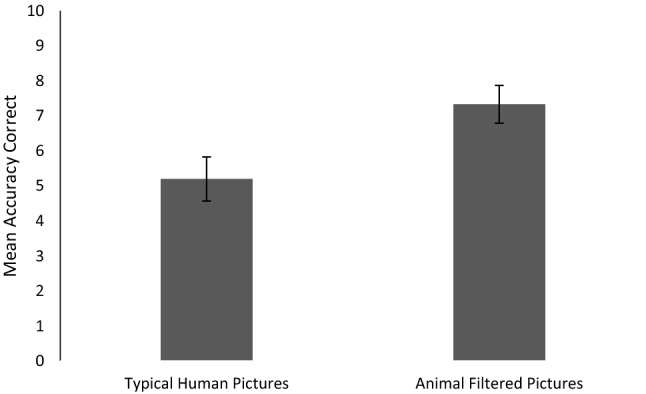
Mean and standard errors of the mean for accuracy correct for each picture set

The data’s distributions (Animal Filter Pictures, SW(15) = .93, p = .273, Original Human Pictures, SW(15) = .943, p = .427, GADS SW(15) = .898, p = .089) did not significantly differ from normality. Data was examined for outliers using box plots in line with recommendations by Field (2017), and none were identified. Matched Samples *t* tests showed that individuals performed the emotion recognition task significantly more accurately with the Animal Filter (M = 7.33, SD = 2.093), than the Original Human pictures (M = 5.2, SD = 2.455), T(14) = 3.506, p = .003, d = 0.92, (please see Fig. 1). A simple linear regression also showed that GADS scores accounted for around 70% of the variance in emotion recognition with Orginal Human pictures (F(1,13) = 49.183, p < .001, R2 = .791, B = .263, Beta = .889, t = 7.01, p < .001), compared to only around 30% with the Animal Filter pictures. (F(1,13) = 6.392, p = .025, R2 = .33, B = .145, Beta = .574, t = 2.528, p = .025.

We also performed exploratory analyses to identify whether the above effects were driven by particular emotions. We performed five separate Fischer’s exact tests analysing hits and misses across the two presentation types (human and animal) for each emotion separately. These analyses suggested that these results were likely driven by greater performance with the animal filtered pictures compared to human for the happy and angry emotions, but not sad, surprised or afraid (see Table 3 for total number of hits and Fischer’s exact values for each emotion type).

**Table 3:** Number of hits for each emotion type split by human versus animal presentation and Fishers exact inferential comparing hits and misses across presentation type

Emotion	Human hits	Animal hits	Fishers exact p values
Happy	16	30	p < .001*
Angry	17	26	p = .02*
Surprised	15	20	p = .295
Afraid	13	17	p = .439
Sad	17	17	p > .99

*p-value is significant at the .05 level

## Discussion

Our results indicate that participants had a 70% success rate when identifying the emotional expressions of faces in the Animal Filter condition, and in contrast had only a 50% success rate in the Original Human condition. Participants’ GADS scores proved to be a much greater indicator of emotion recognition abilities for human rather than animal faces, though both were significantly predictive. Previous research on this topic shows that autistic individuals with mean IQ scores in the average range have improved ToM ability when social agents are non-human rather than human (Atherton and Cross 2018). This study indicates that this pattern persists throughout the wider spectrum, as individuals with autism considered to be low functioning, many of whom also possessed a co-occurring ID also show improved ToM when evaluating non-human versus human agents. Such findings are important as they allow for greater understanding of the similarities of individuals with varying levels of functional ability.

Of interest in this study is to situate results with previous work looking at performance on emotion recognition in individuals with ASD, particularly as those with more severe autism are rarely studied in the context of ToM. For instance, as previously discussed, Sucksmith et al. (2013) found that adults with ASD were impaired when identifying emotions happy, angry, on the KDEF, while those without ASD showed no difference between emotions. Of interest is that in the present study the two emotions driving the effect were also happy and angry, not afraid. In relation to the ability to recognize the animal version of ‘happy’ in our sample, Silva et al. (2015) found that children with ASD showed avoidance towards happy human faces, while approaching happy cartoon faces. Thus, it may be that positive emotions in human faces have different associations for those with ASD which affect identification, and this can be improved when changing characteristics of the stimulus, such as anthropomorphizing them, which makes them more approachable to those with ASD.

Thus, it is clear, in line with the finding that 70% of GADS score variation was explained by the human KDEF measures, that ToM is an important predictor of symptom severity and development (Jones et al. 2018). As this pattern has now been found in autistic people with co-occuring ID, as well as those with average IQ, it would be of future interest to conduct further research into the wider spectrum, and examine whether a similar pattern can be found in those with sub-clinical autistic traits levels. Exploring the stability of this effect independent of IQ may help uncover underlying, shared characteristics of an undeniably diverse group of individuals, thus improving our understanding of the core features of the autism phenotype.

While still speculative, there are several theories as to why autistic people may have differential ToM skills when decoding non-human rather than human faces. While it has been theorized that autistic people may have comparatively reduced social interests (Chevallier et al. 2012), studies such as this one and others instead suggest that social interest in autistic people may fluctuate depending on whether an agent is typically human or non-human. For instance, research shows typical face exploration, including increased attention to eye regions, in autistic samples when faces are presented as animal rather than human (Grandgeorge et al. 2016), and typical neural activation patterns when faces are presented as cartoons rather than humans (Whyte et al. 2016).

The role of motivation in ToM connects with a broader implication of this research, which is that non-human stimuli may be especially rewarding to the autistic population. A large body of work suggests that autistic people have a particular affinity and increased social responsiveness towards animals, and while experiencing anxiety during human contact, this can be ameliorated through animal contact (O’Haire et al. 2013). Examination of neural reward responses patterns indicate that autistic people find it more rewarding to view animal rather than human faces (Whyte et al. 2016).

While autistic people have been theorized to be in less need of social contact than their typically developed (TD) peers, a number of studies rebut this characterization, showing that autistic people report the same degree of social interest as those with TD (Cowart et al. 2004). However, stemming from social differences, autistic people can suffer from exclusion in peer settings (Kasari et al. 2011), and report feeling more lonely than their TD peers (Lasgaard et al. 2010). Thus, it may be possible that a resulting decrease in social self-efficacy causes autistic people to find solace and social reward in non-human agents, which may result in stronger ToM performance when the agent in question is non-human rather than human. With this in mind, it becomes clear that in order to fully understand ToM in autism it is necessary to move beyond the framework of ‘intact’ or ‘impaired.’ Instead, researchers must consider the context in which an autistic person has learned ToM, and how this has shaped their desires or beliefs about mental state processing.

It is important to note the limitations to this study. First, individuals in this study were identified by occupational therapists as having need for significant supports, and were thus educated in special educational settings befitting individuals with severe disability, and exact measures of cognitive ability such as IQ were not obtained. While this opens the interpretation of the analysis to possible confounds regarding heterogeneity in cognitive ability between participants, this is in many ways an unavoidable issue when conducting research on this portion of the ASD population. For instance, research indicates that particularly in individuals with more severe ASD related symptoms, cognitive performance shows peaks and troughs across domains, and there is no specific cognitive profile associated with ASD (Charman et al. 2011). Furthermore, individuals with ASD who also show communicative difficulties are significantly more likely to show poor IQ test performance (Hoekstra et al. 2009). Thus, it can be difficult to directly test individuals with severe ASD symptoms, such as those in this sample, in a way that reveals a true IQ score independent of communicative impairment and reflects the variability in performance across various subscales. For this reason, it was decided to instead utilize the assessments of on-site specialists with an in-depth knowledge of the participants to situate their developmental functioning within the broader autism spectrum. Additionally, it would be of interest to explore visual saccades and obtain more detailed data from eye tracking in relation to face recognition in ASD, specifically comparing visual saccade patterns when viewing human and animal faces.

Despite these limitations, this study examined ToM ability not only with regard to stimulus type, but also to devise a means to test a portion of the autism population that is not typically assessed on ToM. For instance, as discussed by Jarrold and Brock (2004), in order to isolate the specific domain of impairment relative to controls, the majority of autism research is conducted on people with autism who also have average to above average IQ and whose developmental age matches their chronological age. The limitation therein is that those with lower developmental ages, IQ scores and who possess co-occurring intellectual disabilities are not represented in autism research, and findings can not necessarily be generalized to such individuals. Thus, in this study we demonstrate a way to simplify the KDEF testing process, thus allowing for greater inclusion in facial recognition tests for those with autism, and shedding light on possible ToM mechanisms for a greater proportion of the autism spectrum.
